# The Role of Occupational Therapy in the Care of Informal Care Partners of People Living with Dementia: A Concept Analysis

**DOI:** 10.1002/alz70858_101684

**Published:** 2025-12-25

**Authors:** Ashley M Taylor, Steven Gerardi, Elizabeth DeLuca‐Berg

**Affiliations:** ^1^ University of St. Augustine for Health Sciences, Austin, TX, USA

## Abstract

**Background:**

*Informal caregivers* (care partners) are people, such as family members, who provide care to those who cannot care for themselves, including people with dementia (PwD). Care partners assist PwD in their daily occupations, often without training or guidance from healthcare professionals. Informal care partners typically balance multiple roles, such as caregiver, parent, spouse, and worker, which can become increasingly difficult as the disease progresses. Caring for PwD profoundly impacts care partners and can present a range of emotional, physical, financial, and occupational challenges. Occupational therapy (OT) has an important role to play in the care of PwD. However, when treating PwD, if clinicians do not operate from a dyadic model, the needs of the care partners may be overlooked. Although addressing both components of the care partner dyad is important to the delivery of OT services, the role of OT in the care of informal caregivers of PwD is not well articulated in the OT literature, which makes it difficult for OT practitioners to fill this role. As such, conveying the role of OT in the care of care partners can improve the delivery of OT services to PwD.

**Methods:**

Walker and Avant's eight‐step approach to conduct a concept analysis was used to assess and clarify the role of OT in the care of informal caregivers of PwD.

**Results:**

The literature on OT with caregivers of PwD was reviewed, which yielded data on the OT treatment of the care partner dyad. Informed by these findings, and the OT practice framework, the defining attributes of the role of OT in the care of informal caregiver of PwD were determined. The model of human occupation was used to frame a model case. A contrary case was described, as well as the attributes of OT that frame its role in caring for the care partner dyad of PwD.

**Conclusions:**

The role of OT with informal caregivers of PwD is to identify barriers to participation and challenges to effective occupational performance, and to implement interventions to enable participation and enhance performance. By addressing the care partner dyad, better OT services can be provided.

